# Populous: a tool for building OWL ontologies from templates

**DOI:** 10.1186/1471-2105-13-S1-S5

**Published:** 2012-01-25

**Authors:** Simon Jupp, Matthew Horridge, Luigi Iannone, Julie Klein, Stuart Owen, Joost Schanstra, Katy Wolstencroft, Robert Stevens

**Affiliations:** 1 Bio-Health Informatics Group, School of Computer Science, Kilburn Building, Oxford Road, Manchester, UK, M13 9PL; 2 Inserm U1048, Institute of Metabolic and Cardiovascular Diseases - I2MC, 1 avenue Jean Poulhés, B.P. 84225, 31432 Toulouse Cedex 4, France

## Abstract

**Background:**

Ontologies are being developed for the life sciences to standardise the way we describe and interpret the wealth of data currently being generated. As more ontology based applications begin to emerge, tools are required that enable domain experts to contribute their knowledge to the growing pool of ontologies. There are many barriers that prevent domain experts engaging in the ontology development process and novel tools are needed to break down these barriers to engage a wider community of scientists.

**Results:**

We present Populous, a tool for gathering content with which to construct an ontology. Domain experts need to add content, that is often repetitive in its form, but without having to tackle the underlying ontological representation. Populous presents users with a table based form in which columns are constrained to take values from particular ontologies. Populated tables are mapped to patterns that can then be used to automatically generate the ontology's content. These forms can be exported as spreadsheets, providing an interface that is much more familiar to many biologists.

**Conclusions:**

Populous's contribution is in the knowledge gathering stage of ontology development; it separates knowledge gathering from the conceptualisation and axiomatisation, as well as separating the user from the standard ontology authoring environments. Populous is by no means a replacement for standard ontology editing tools, but instead provides a useful platform for engaging a wider community of scientists in the mass production of ontology content.

## Background

The increasing quantity of bio-medical data being published in both the databases and the literature provides many challenges for bioinformatics analysis. The integration and analysis of these data can benefit from rich, standardised meta-data that enable humans and computer applications to give some level of meaning to those data in order to interpret those data appropriately. The development and adoption of such standards is, however, both time consuming and costly. Many bio-medical ontologies are under development to provide reference vocabularies that aim to standardised the way bio-medical data are described [1-3]. In addition to providing the concepts of the domain, these ontologies provide details of the relationships between domain concepts. These relationships have well defined semantics that facilitate reasoning and consistency checking over the data.

There are now many bio-medical ontologies that are well developed and in regular use across the discipline [1,4]. In this paper we present the Populous application that provides a framework within which domain experts can contribute their knowledge to a developing ontology. Populous uses a simple template based approach to ontology construction, but with semantic constraints that guide the filling of those templates; we exemplify its use in the development of an application ontology for the Kidney and Urinary Pathway (KUP) domain.

In the life sciences, efforts such as the OBO foundry [1] aim to provide the domain with a set of orthogonal interoperable reference ontologies. Within the OBO foundry there are a core set of ontologies that cover different domains ranging from genes, proteins and chemical entities, through to cells, anatomy and phenotype ontologies. These ontologies, along with many others that sit outside the OBO foundry, provide a set of 'building blocks' for building new application specific ontologies [5]. Re-using modules from existing ontologies to build larger and more complex compositional ontologies lowers the cost of development and maintenance. In addition, it offers greater opportunities for data integration and data interoperation in applications that exploit those ontologies. The Gene Ontology (GO) consortium has recently released guidelines for the development of so-called cross product ontologies that allow concepts from one OBO ontology to be *composed *or described in terms of concepts from other OBO ontologies [6]. These rich conceptualisations offer many benefits in terms of querying and reasoning over data described by these ontologies [5,7,8]. This modular approach to developing ontologies is based on an ontology design pattern known as normalisation [9]. As in software engineering, design patterns are based on good practices and are a useful tool for developers. They provide templates that act as guidelines to ease development in large collaborative projects [10-13]. Identifying suitable design patterns is hard, however, once a pattern is established, population of the pattern can occur rapidly. In this context we define *population *as the creation of multiple instances of a particular design pattern of axioms, that can include classes and individuals in an ontology.

We can break down the pattern based development process into a series of steps:

1. Creation of an ontological framework that establishes the patterns of axiomatisation that will need 'populating';

2. Identifying the design patterns that capture some aspect of the ontology's domain;

3. Creating a template for that pattern that can be populated by the ontology's author;

4. Filling the template according to the pattern;

5. Transforming the content of the template into instances of the pattern;

6. Placing the instantiated pattern into the final ontology.

Steps 1 and 2 are hard, and requires ontology design skills, knowledge of the ontology in question, the principles and style of the ontology, and the ontology engineering process. The remaining steps require appropriate tool support to assist developers in populating and applying the pattern. Modern ontology editors, such as OBO edit [14] or Protégé [15], offer a wide range of support for building ontologies by hand, but offer less in the way of support for modelling design patterns and populating templates. In addition these tools require training and can be overwhelming for domain experts who are new to ontology building. To address this issue we developed the Populous application that supports steps 3-6 and we demonstrate how it has been used by domain experts to populate ontology design patterns *en mass*.

### Related work

Developing ontologies according to some design pattern is not a novel concept and is considered good practice for large ontology development projects [10-13]. As an example of a pattern, consider an ontology about cells; eukaryotic cells can be classified as being either anucleate, mono-nucleate, binucleate or multinucleate. We can abstract over this pattern to say that every cell can be classified by its nucleation. This pattern is repeated for all cell types; the only variables are the cell name and the value for its nucleation. We can create a simple template for this pattern that could be populated by a cytologist, without him or her needing to worry about the underlying ontological representation.

Building ontologies from templates allows abstraction over the underlying design patterns. A tabular layout provides a simple and intuitive form fill-in style of user interface that can support the population of such templates. Each row can correspond to a member from a set of related entities and each column represents the type of relationship. The intersection of row and column holds the 'filler' for the given entity's relationship of that column's type. By adopting such templates, ontology developers can separate the pattern from its instantiation; this allows the domain expert to focus on the knowledge without the distraction of a knowledge representation language.

Templates are useful when data, information or knowledge need to be collected in a regular form. Applying constraints to the template reduces the number of discrepancies in the input data. A common tool for collecting data in this form is the spreadsheet; spreadsheets provide a tabular interface, where columns and rows represent certain attributes, and individual cells capture the data. Tables help users to structure data in a logical way, that is useful for both its maintenance and processing. In ontology development, spreadsheets can be used to gather and organise information about concepts and their relationships.

Previous work in this area has focused on the transformation of data into ontologies, but little attention has been paid to supporting the population of the templates at the point of data entry and this is where Populous's main contribution lies.

Various tools are available to support the conversion of spreadsheet data into statements in a knowledge representation language. Excel2RDF [16], Convert2RDF [17], and RDF123 [18] are three tools that allow users to generate Resource Description Framework (RDF) statements from spreadsheets. Despite RDF being the reference syntax for the Web Ontology Language (OWL), its serialisation is complex and not intended for humans, making it inappropriate for defining higher level OWL constructs in patterns.

The ExcelImporter plugin [19] for Protégé 4.0 was a step up from these tools and enabled users to transform spreadsheet content directly into OWL axioms. It was, however, limited to only a small set of OWL constructs. The more recent tools to support template data and pattern instantiation include Mapping Master [20], OPPL 2 [12,21] and the Protégé Matrix plugin [22].

• The MappingMaster plugin for the Protégé 3.4 ontology editor is a more flexible tool for transforming arbitrary spreadsheet data into OWL. MappingMaster moves away from the row centric view of spreadsheets and has an expressive macro language called M^2 ^[20,23] that can handle non-uniform and complex spreadsheets. M^2 ^combines a macro language for referring to cells in a spreadsheet with a human readable syntax for generating OWL expressions called the Manchester OWL Syntax [24]. MappingMaster and M^2 ^are primarily designed for the transformation of spreadsheet data to OWL, but provides little in the way of support for populating and validating the spreadsheet data.

• The Ontology Pre-Processor Language (OPPL) [12,21] (version 2) [25] is a scripting language similar to M^2^. OPPL 2 is also Manchester OWL Syntax based and allows for the manipulation of OWL ontologies at the axiom level. OPPL 2 has support for the use of variables and the addition and removal of logical axioms from an ontology. OPPL 2 is a powerful scripting language for OWL and a user interface is provided via the OPPL plugin for Protégé 4.1 along with a standalone API to embed it into software systems. OPPL 2 does not currently support working with tabular data and is decoupled from any knowledge gathering.

• The MatrixPlugin for Protégé 4.0 allows users to specify simple OWL patterns in a tabular interface that can be used to populate repeating patterns with existing concepts from an ontology. This plugin is useful for ontology developers that have repetitive patterns to instantiate, and has the added benefit of cell validation and auto-completion at the point of data entry. The Matrix plugin is limited by the type of patterns that can be expressed along with the fact that it is tightly integrated with the Protégé interface, therefore, not suitable for all users. It does, however, combine knowledge gathering and axiom generation.

## Results

In order to evaluate Populous in a real ontology building scenario it has been used to populate a template for gathering knowledge about the kidney and urinary system. The kidney is a complex organ composed of several distinct anatomical compartments that together enable the filtration of waste from the blood in the form of urine. Each of the kidney compartments is formed from a wide variety of cell types, and the specificity of the compartments relies on these specialised cell functions. The Kidney and Urinary Pathway Ontology (KUPO) [8] describes kidney cells, their function and their anatomical locations. KUPO is being built to annotate and integrate multi-omics datasets held in the Kidney and Urinary Pathway Knowledge Base (KUPKB) [26].

**Figure 1:**
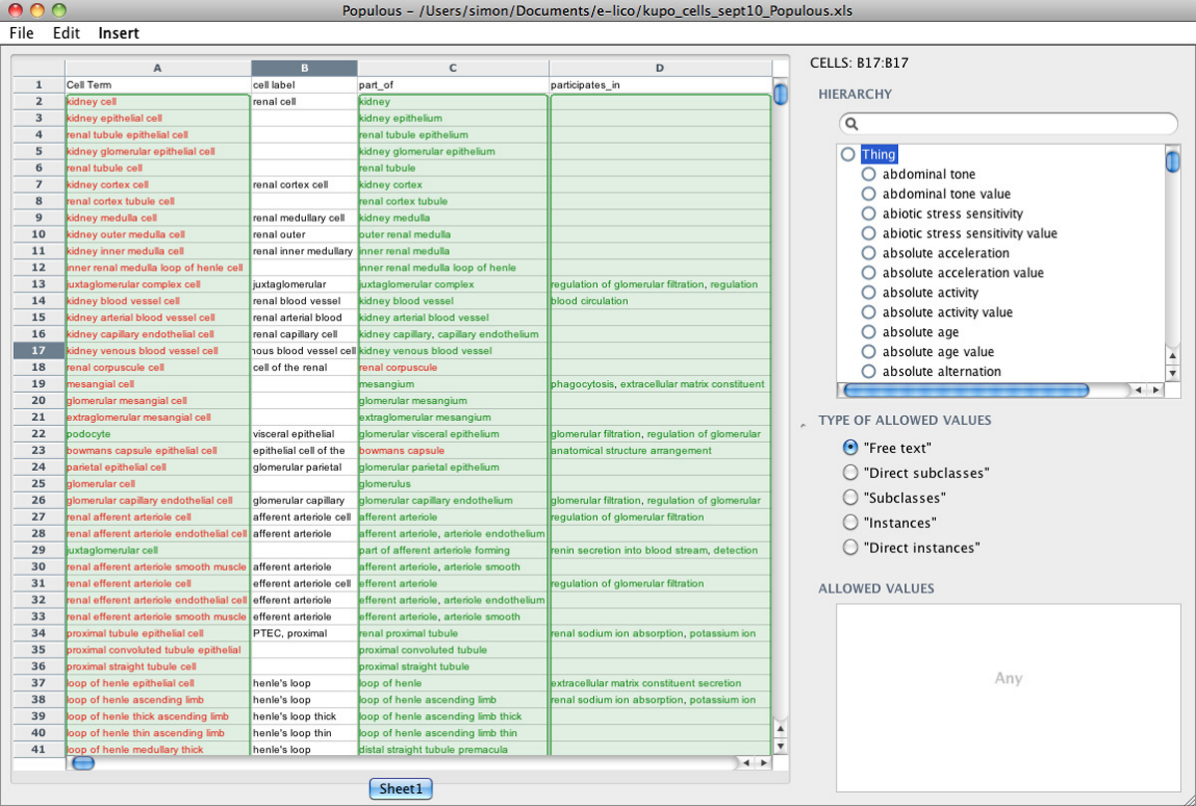
**Populous interface**. Screenshot of Populous showing template population for KUP ontology.

A simple template was designed for experts from the KUP domain to capture the relationships between cell types, their anatomical location and their biological functions. The template has three main columns; column A is for entering cell type terms, column C is for anatomy terms and column D for biological process terms. Populous was used to constrain the allowable values in columns A, C and D to concepts from the Open Biomedical Ontology Cell Type Ontology [27], subclasses or part of the *Kidney *or *Urinary system *concepts from the Mouse Adult Gross Anatomy Ontology [28], and all subclasses of the *Biological Process *concept from the Gene Ontology [29], respectively. The experts were instructed that the relationship between concepts in column A and C was *part of*, and the relationship between column A and D, *participates in*. For concepts that were related to multiple concepts they were allowed to list concepts in a cell separated by a vertical bar. Figure 1 is a screen shot of Populous populated with data from the domain experts.

In order to transform the tabular data into an OWL representation the OPPL patterns in example 1 and 2 were created by the ontology engineers. These patterns state that a cell type is equivalent to a cell that is *part of *an anatomy term and a subclass of cells that *participate in *a biological processes; we use the relationships from the OBO Relations Ontology [30] where appropriate. For both restrictions the existential (*some*) quantification is used. We put aside any ontological issues about our choice of modelling at this point as these can be altered later down the line in the development process. The two differentia in this pattern for a cell genus are the anatomical location and the biological process, which is retrieved from column A, C and D respectively in the template. The entire KUP ontology is generated from the template data combined with the ontology pattern. Example 3 shows the Manchester OWL syntax generated from the pattern and data from row 13 for the *Juxtaglomerular complex cell*.

OPPL example 1: OPPL 2 patterns for describing cell types in terms of anatomy

?cell:CLASS,

?anatomyPart:CLASS,

?partOfRestriction:CLASS = cell and ro:part_of some ?anatomyPart,

?anatomyIntersection:CLASS = createIntersection(?partOfRestriction.VALUES)

BEGIN

ADD ?cell equivalentTo ?anatomyIntersection

END;

OPPL example 2: OPPL 2 patterns for describing cell types in terms of biological process

?cell:CLASS,

?participant:CLASS,

?participatesRestriction:CLASS = ?cell and ro:participates_in some ?participant,

?participatesIntersection:CLASS = createIntersection(?participatesRestriction.VALUES)

BEGIN

ADD ?cell SubClassOf ?participatesIntersection

END;

Example 3: Manchester OWL syntax for Juxtaglomerular complex cell (MA_0002546 = 'part of afferent arteriole forming juxtaglomerular complex', GO_0003093 = 'regulation of glomerular filtration' and GO_0003098 = 'tubuloglomerular feedback' and GO_0003106 = 'regulation of glomerular filtration by angiotensin')

Class: kupo:KUPO_0001028

EquivalentTo:

cell:CL_0000000

and (ro:part_f some MA:MA_0002546)

SubClassOf:

cell:CL_0000000,

ro:participates_in some gene_ontology:GO_0003093,

ro:participates_in some gene_ontology:GO_0003098,

ro:participates_in some gene_ontology:GO_0003106

In addition to the KUPO, additional ontologies were needed to annotate the experimental data. These included ontologies to describe the experimental protocols, experimental factors, and the different animal models under investigation along, with a host of renal diseases. For each fragment of the ontology different templates were generated to be populated in Populous by the domain experts. For each template we strived to re-use concepts from external ontologies such as the disease ontology [31], the experimental factor ontology [5], the ontology of biomedical investigation [7] and the phenotype ontology [32]. Again, by exposing the renal biologists to these reference ontologies through Populous, they were able to provide useful insights about those ontologies. On several occasions they found that key domain concepts were either missing or had been inappropriately labelled [33].

**Figure 2:**
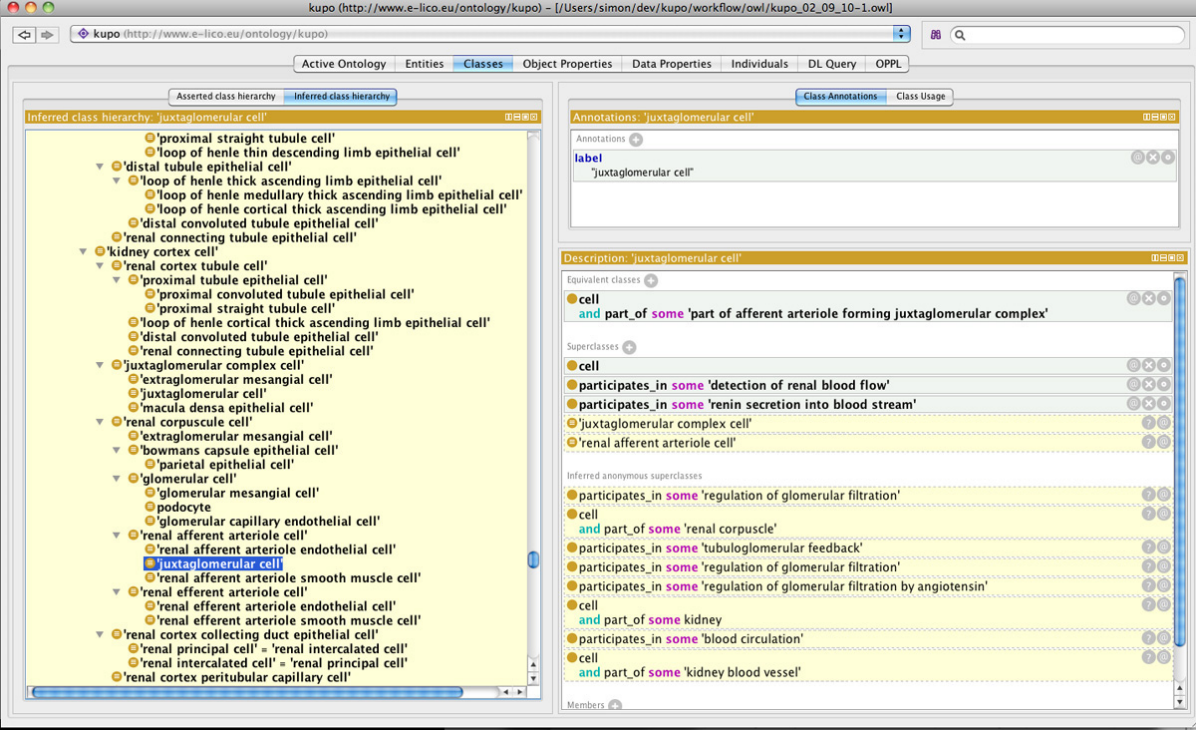
**KUPO in Protégé**. Screenshot of KUPO loaded into Protégé 4.1 showing inferred class hierarchy for Juxtaglomerular cell.

Using this template approach, the domain experts described over 190 cell types, many of which are absent from the current cell type ontology (CTO), along with a further 800 classes that were added to describe the various experimental metadata. Figure 2 shows a section of the inferred hierarchy after classifying the ontology in Protégé 4.1. Cell classes are asserted without hierarchy and form a flat list. The partonomy of the mouse anatomy is used to drive inferences about super/sub class relationships between cell types.

Leaving the reasoner to compute the class hierarchy means the domain experts can manually inspect for missing or incorrect subsumptions. In cases where a desirable subsumption relationships could not be achieved using a partonomic relation, users were free to assert child/parent relationships in another column. This methodology provided a useful feedback system between the domain expert and the ontologist developing the design patterns. Using this approach the domain experts were able to focus on the biological knowledge and allow the ontologist, who was not an expert in the domain, to focus on the conceptualisation.

## Discussion

Populous is designed for domain experts to gather knowledge that can be subsequently used to build ontologies. Whilst previous tools have provided support for transforming templates into ontologies, they lacked basic support to help the user at the point of data entry and knowledge gathering. Populous was designed to fill this niche and meets the requirements outlined in the Method section. The simple tabular interface used in Populous is familiar to users who have already used a spreadsheet application. This lightweight interface offers a way to engage domain experts in the ontology authoring process without issue trackers, face to face meetings and so on. Such mechanisms, however, remain a valuable part of ontology development.

We have demonstrated how Populous can be used to develop an ontology describing cells of the kidney and urinary pathway. This demonstration highlights how domain experts managed to generate a real application ontology without being exposed to an ontology language like OWL, or a sophisticated ontology editor like Protégé. Populous's main purpose is for knowledge gathering and not ontologising. By shielding users from the ontology, except for review later in the process, they are left to concentrate on the biology and not worry about the axioms needed to represent it. The 'ontologising' needs to happen, but it happens at a different stage of the process by someone with the 'ontologising' role. This separation is particularly useful should the ontologist wish to change the conceptualisation or experiment with different patterns for the representation.

Our experience in developing the KUPO with Populous provided some insights into the benefits of developing an ontology in this way. Classical approaches to ontology development have tended to focus on organising domain concepts into hierarchies. The approach used for KUPO shifts the focus from the hierarchy and allows us to focus on the relationships that describe those entities. By axiomatising these relationships through our design patterns we can exploit the reasoner to manage any hierarchical classification. Using the reasoner to compute subsumptions facilitates logical explanations as to why certain relationships hold. We also see how building modular ontologies in this way encourages the domain experts to contribute their expertise to the external ontologies they might be using. For example, there are renal cell types for the vasa-recta descending limb and the vasa-recta ascending limb, both of which have different functions. The domain experts wanted to distinguish between these two cell types according to their anatomical location, however, the mouse anatomy ontology only describes the vasa-recta. The domain experts spotted this omission in the mouse anatomy ontology and were able to feed this back to the developers. Building modular and normalised ontologies is considered a good ontology engineering practice [9], however few existing bio-medical ontologies are built in this way. We have shown in the development of KUPO that Populous encourages and supports the development of ontologies in this way.

The question now arises as to how far can you go with a tool like Populous? Populous is by no means a replacement for full blown ontology editors, nor is it intended to be. Existing tools provide the means to create an ontology development framework, within which Populous would have a role. The framework would include patterns that have been developed to model the ontology's domain. As Populous is used to instantiate these patterns and build the ontology any changes to the underlying framework can happen independently of the efforts by the domain experts. Developing good design patterns up front can be difficult, so it is important that whichever framework is adopted can readily accommodate changes in how the domain should be modelled. In the KUPO development, such an extension to our initial framework was required. An early naive assumption was that all kidney cells could be described in terms of their anatomy alone, only to later find some exceptions to this assumption. For example, renal principal and renal intercalated cells are currently indistinguishable by anatomy and function alone. In these cases we can add new patterns, such as the ability to describe a cell in terms of its lineage. Such an extension is trivial in Populous, as we can simply add a new column for the relationship, and a new OPPL pattern to handle the axiomatisation.

The template approach can be particularly advantageous in scenarios where the modelling needs to change. Peters *et al *[34] showed how templates can be used to generate different ontological representations of the same data. The KUPO is being used to annotate data in the KUPKB. The KUPKB is an RDF triple store, thus only a limited set of OWL inferences are possible. Querying complex OWL ontologies in a triple store with a language like SPARQL can be cumbersome, so an alternate representation of the KUPO data may be more suitable. Generating a simpler representation of the KUPO in Populous is possible by replacing only the OPPL patterns. This is the case so long as the classes or instances in the patterns do not change; if they do, then the knowledge gathered has to changed and the process starts again, but again the separation of knowledge gathering and knowledge generation helps this process.

### Future work

The release of Populous as presented is an early version; there remains many possible additions. OPPL 2 provides an expressive language for generating patterns that include all constructs from the OWL 2 specification. OPPL's support for variables make mapping columns from tabular data to variables both flexible and convenient. OPPL's built in macro extensions enable the dynamic expansions of OWL expressions. For example, we can create a conjugation of OWL expressions from a set of values assigned to a single OPPL variable. We see a potential limitation of Populous as it assumes a row-per-entity paradigm where single columns map to a particular variable. This layout structure is simple but may not be suitable for all types of conceivable template. Fortunately, the M^2 ^language has been specifically designed to work with these kinds of spreadsheets and it offers many complementary functions to a language like OPPL. All templates populated in Populous can be saved as Micorsoft Excel files and loaded into the MappingMaster plugin should users wish to transform them into OWL using M^2^.

Other potential future additions include:

1. Populous allows multiple values to be entered in a cell using the vertical bar separator. This syntax is used to define a value set for a particular OPPL variable. These value sets are subsequently used by OPPL to dynamically create conjunctions of OWL expressions that contain a mapping to that variable. Future extensions to the Populous syntax will give the user more flexibility when asserting value sets, such as the ability to state whether the relationships represent an intersection or a union of variables.

2. Populous currently gathers domain knowledge for the ontology, but not about the ontology. We aim to extend Populous to support various metadata such as editorial metadata and definitional metadata etc.

3. Populous is a single user application. Making Populous collaborative such that contributors may collectively add material to the same spreadsheet.

4. Feedback from the generated ontology to fix or extend data in Populous is currently *ad hoc*. A tighter coupling of this feedback cycle, without having to go into an axiom based editor, will increase the quality assurance aspects of Populous.

## Conclusion

Populous offers a means of creating ontology content without the use of a standard ontology development tool. We see Populous as an extension to the current set of ontology development tools that offers a new avenue for engaging domain experts in the ontology development process. It is possible to separate knowledge gathering from conceptualisation and axiomatisation and Populous is one means of achieving this goal. Such a separation offers flexibility and the simple form fill-in style of knowledge gathering should make generation of axiomatically rich ontologies increasingly straight-forward.

## Implementation

### Requirements analysis

All of the previous tools developed in this area tend to focus on the transformation from the template to the ontology. They provide little or no support for populating and validating template content.

Furthermore, tools like ExcelImporter, the OPPL Plugin and MappingMaster are integrated into the ontology development tools, that can be overwhelming to users new to ontology development. We wanted to explore the use of a simpler tabular based interface to ontology authoring that shields the user from the underlying ontology and guides them when populating the template. Providing validation at the time of authorship should significantly reduce the amount of time required to debug and process the data captured in the spreadsheet. Here we list some key requirements for a tabular based ontology building tool:

1. New concepts may be created or reused from other ontologies when populating the template. In setting up Populous the users must be able to load and browse ontologies or parts of ontologies, that form part of the ontology being developed.

2. The set of valid concepts allowed in a particular column may be constrained to concepts from other ontologies, or parts of ontologies. Each time a concept is added to a cell within that column the value is validated according to the constraint.

3. To improve human comprehension the concept should be rendered using only the URI fragment, or optionally a human readable label from the ontology.

4. A table cell might have multiple values; for example, when the concept being described has multiple parts.

5. Users should be free to suggest new concepts when an appropriate concept is not available.

### Populous

Populous is an extension of RightField [35], a tool that has been developed to support ontology building from spreadsheets; RightField is for creating Excel documents that contain ontology based restrictions on a spreadsheet's content. In RightField a user can open Excel spreadsheets and ontologies from their local file systems or from the BioPortal [36,37]. RightField can read OWL, OBO and RDFS ontologies. Using RightField, individual cells, or whole columns or rows can be marked with the required ranges of ontology terms. For example, they could include all subclasses from a chosen class, direct subclasses only, all individuals, or only direct individuals. Each spreadsheet can be annotated with terms from multiple ontologies. RightField is primarily designed for generating spreadsheet templates for data annotation; Populous extends RightField to support knowledge gathering and ontology generation. Populous builds on top of the RightField machinery for embedding ontology terms into spreadsheet cells and provides support for transformation of these spreadsheets into OWL ontologies.

Populous and RightField are both open source cross platform Java applications. They use the Apache-POI [38] for interacting with Microsoft documents and manipulating Excel spreadsheets. Populous is available for download from http://www.populous.org.uk.

Requirement 1 is already addressed using RightField functionality to upload both OWL and OBO ontologies. In order to better serve the life science community, users can also browse and load ontologies directly from BioPortal. Once the ontologies are loaded they are classified by a reasoner and the basic class hierarchy can be viewed.

Requirement 2 is met by the ability to select terms from the ontology to create *validation sets*. A data validation restricts the set of values that are valid for a particular cell, or selection of cells, in the table. Validations can span multiple rows and columns and be composed of all classes from a specified ontology, or can be further restricted to subsets of classes, properties or individuals from a chosen ontology. These data validations are stored in hidden worksheets along with additional information such as the full URI for the term, a label and the source ontology URI. These templates can also be exported as Microsoft Excel documents, which preserve the data validations placed on the cells, and can be opened in application that supports the 1997-2004 Microsoft Excel (.xls) file type.

We address requirement 3 by allowing users to populate cells using ontology labels. Once data has been entered the default will be to render the ontology term using its label; if no label is specified the URI fragment is used. RightField already supports reading Excel workbooks, so users are free to populate the templates in external tools before importing them into Populous for validation and transformation.

By using Populous directly users will benefit from having instant validation of the input data, satisfying requirement 2, along with some advanced features such as regular expression based auto-completion as they type into annotated cells. Additionally Populous supports the addition of multiple values into a single cell that are validated individually according to requirement 4. This can be particularly useful for certain kinds of patterns where a conjunction of variables is required to construct the axiom (See OPPL examples 1 and 2 in Results section). Populous also allows the addition of free text values, even if the cell has an associated validation range, thus satisfying requirement 5. These values are highlighted to the user in red and can act as placeholders for new or suggested terms when no suitable candidate could be found in the validation set. Populous supports the use of OPPL 2 patterns in order to generate new OWL axioms from the populated template. OPPL 2 scripts can be written directly in Populous's design mode or imported from scripts generated in the OPPL plugin for Protégé. Variables from the OPPL pattern must be mapped to columns from the table using the column name. A pattern Wizard guides the user through the generation and execution of the OPPL scripts. When the template is processed new identifiers for unknown terms can be auto-generated and exported from Populous.

### Building an ontology with Populous

**Figure 3:**
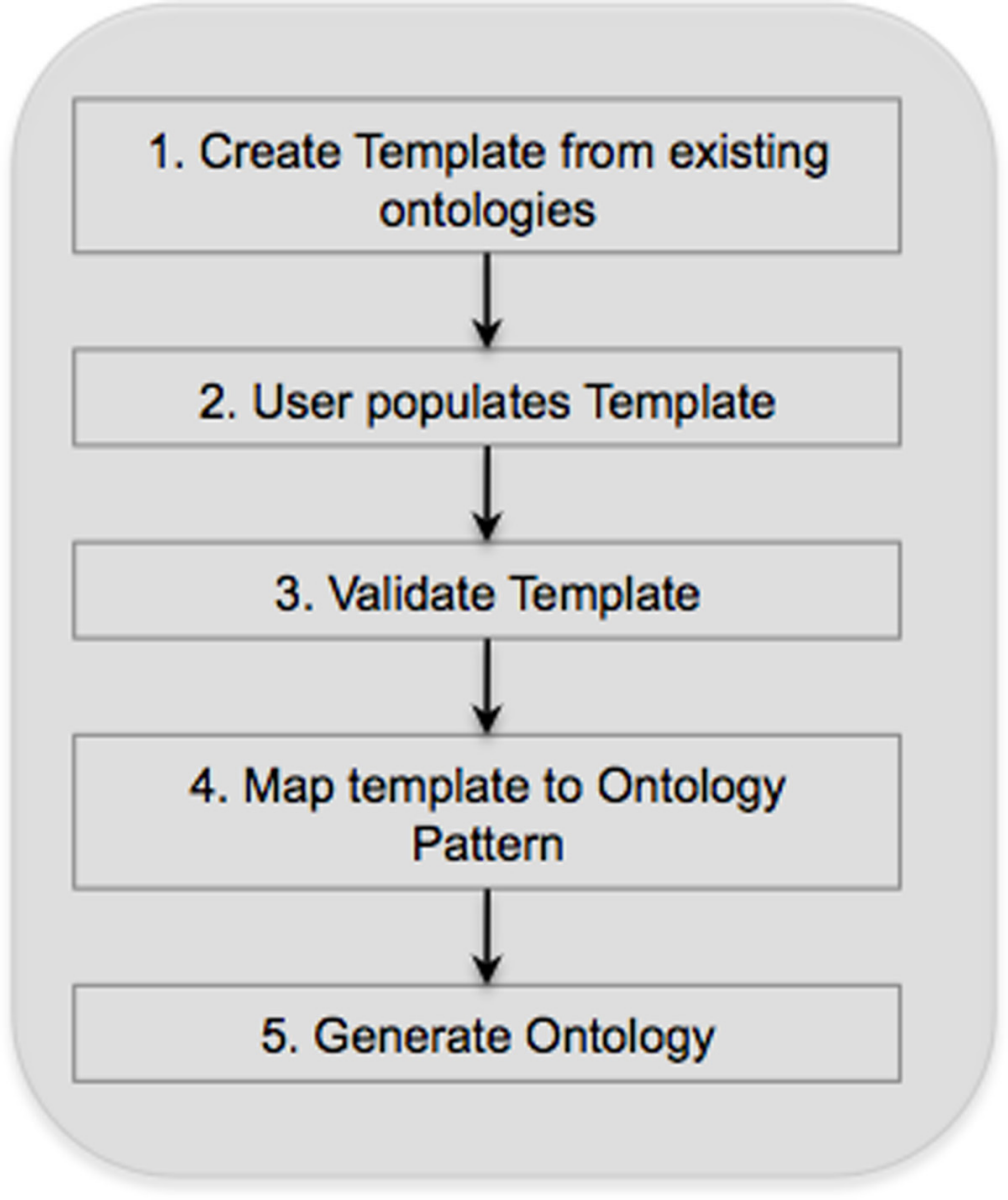
**Ontology generation workflow**. Outline of typical workflow for ontology generation with Populous.

A typical workflow for building ontologies with Populous is depicted in Figure 3. We can demonstrate Populous in building a simple ontology using the cell type nucleation example described in the introduction. The pattern in the ontology states that every cell must have a nucleation. We need to create a template with two columns, column A is for cell type concepts, whilst column B is for nucleation concepts. Ontologies describing cells and their nucleation already exist that we can import into Populous. By connecting to BioPortal we can load the Cell Type Ontology (CTO) [27] and the Phenotype and Trait Ontology (PATO) [32]. In order to restrict column A to terms from the CTO, we highlight all the cells in column A and restrict them to all subclasses of the root class. Column B is restricted to subclasses of the *nucleation *concept from PATO. The template is now ready to be populated by the domain expert.

**Figure 4:**
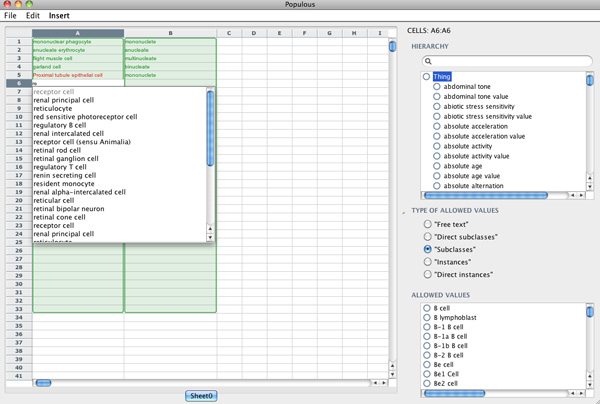
**Populous template for cell types**. Screenshot of Populous showing template population for cell types and nucleation.

Figure 4 shows a partly populated template. The terms in green indicate a valid term has been entered into the cell. The term in Column A5, *Proximal tubule epithelial cell *is red because it is not a valid term from the CTO. Cell A6 is in the process of being edited with the auto-completer, which offers a valid suggestion for input.

**Figure 5:**
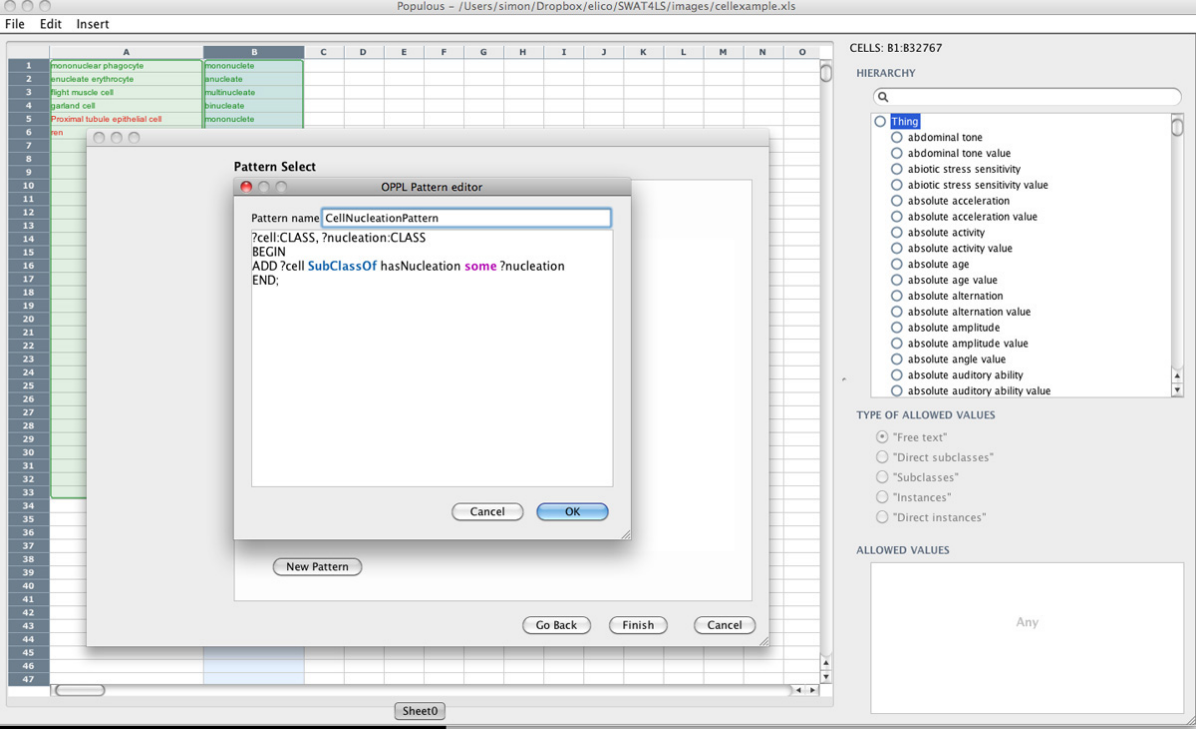
**Populous OPPL wizard**. Screenshot of Populous Pattern Wizard showing the OPPL script editor.

The populated spreadsheet can now be transformed into an ontology. This can be done using the pattern wizard in Populous (Figure 5). The first step in the pattern wizard asks the user to select the columns and rows that contain populated data. In this example the pattern creates a restriction on each cell stating that all cells have a relationship, called *hasNucleation*, to an instance of the class nucleation This pattern can be expressed in OPPL 2 using the following example.

Example 4: OPPL 2 pattern for cells and nucleation

?cell:CLASS,

?nucleation:CLASS

BEGIN

ADD ?cell SubClassOf hasNucleation some ?nucleation

END;

There are two variables in the pattern, ?cell and ?nucleation. These variables are mapped to column A and B respectively. The pattern is to be instantiated using data from rows one to six that must be specified in the Wizard. The next step involves validating the pattern, given that *Proximal tubule epithelial cell *is unknown by the validator, the user is given the option to assign a new URI for this concept. The final step involves specifying the full OPPL pattern needed to generate the OWL axioms. The workflow specified using the wizard can be saved and re-loaded for future re-use. The OPPL wizard provides support for managing how new concepts are dealt with, including a flexible mechanism for generating new URIs and dealing with concept annotations. Each axiom generated by OPPL is added to the ontology with an associated annotation, that helps track the provenance of the generated axioms. Once the wizard is complete the OPPL is executed over the spreadsheet and the resulting generated OWL ontology is displayed to the user in Manchester OWL syntax. A copy of the ontology is saved to disc in RDF/XML, although other OWL syntaxes are available. Example 5 shows the Manchester syntax generated for this example. A complete grammar for the OPPL 2 syntax is available at http://oppl2.sourceforge.net/grammar.html and further documentation is available at http://oppl2.sourceforge.net/taggedexamples.

Example 5: Mononuclear Phagocyte described in Manchester OWL syntax generated from the OPPL 2 pattern in Example 4

(*PATO_0001407 is the identifier for *mononucleate)

Class: cto:CL_0000113

SubClassOf:

hasNucleation some pato:PATO_0001407

## Abbreviations

API: Application Programming Interface; CTO: Cell Type Ontology; GO: Gene Ontology; KUP: Kidney and Urinary Pathway; KUPO: Kidney and Urinary Pathway Ontology; KUPKB: Kidney and Urinary Pathway Knowledge Base; MAO: Mouse Adult Gross Anatomy Ontology; OBO: Open Biomedical Ontologies; OPPL: Ontology Pre-Processing Language; OWL: Web Ontology Language; PATO: Phenotypic Quality Ontology; RDF: Resource Description Framework; RDFS: Resource Description Framework Schema; SPARQL: SPARQL Protocol and RDF Query Language; URI: Uniform Resource Identifier; XML: Extensible Markup Language.

## Competing interests

The authors declare that they have no competing interests.
